# Embodied Semantics in a Second Language: Critical Review and Clinical Implications

**DOI:** 10.3389/fnhum.2019.00110

**Published:** 2019-03-29

**Authors:** Elisa Monaco, Lea B. Jost, Pascal M. Gygax, Jean-Marie Annoni

**Affiliations:** ^1^Laboratory for Cognitive and Neurological Sciences, Neurology Unit, Medicine Section, Department of Neuroscience and Movement Science, Faculty of Science and Medicine, University of Fribourg, Fribourg, Switzerland; ^2^Department of Psychology, University of Fribourg, Fribourg, Switzerland; ^3^Neurology Unit, Fribourg Cantonal Hospital, Fribourg, Switzerland

**Keywords:** aphasia, bilingualism, clinical rehabilitation, embodiment, semantics

## Abstract

The role of the sensorimotor system in second language (L2) semantic processing as well as its clinical implications for bilingual patients has hitherto been neglected. We offer an overview of the issues at stake in this under-investigated field, presenting the theoretical and clinical relevance of studying L2 embodiment and reviewing the few studies on this topic. We highlight that (a) the sensorimotor network is involved in L2 processing, and that (b) in most studies, L2 is differently embodied than L1, reflected in a lower degree or in a different pattern of L2 embodiment. Importantly, we outline critical issues to be addressed in order to guide future research. We also delineate the subsequent steps needed to confirm or dismiss the value of language therapeutic approaches based on embodiment theories as a complement of speech and language therapies in adult bilinguals.

## Introduction

The term “embodiment” refers to the grounding of cognition in systems involved in low level perceptual and action information processing. Embodied theories of cognition claim that higher cognitive processing, including language, activates the same brain sensorimotor structures involved when experiencing the environment (e.g., Glenberg, 1997; Glenberg and Kaschak, 2002; Gallese and Lakoff, 2005; Pulvermüller et al., 2005; Barsalou, 2008; Jirak et al., 2010; Meteyard et al., 2012). Converging clinical and neurophysiological evidence indicates that semantic knowledge is grounded in different heteromodal but also on modality specific cortical regions, coding for perceptual, sensory, visual, auditory, motor or affective experiential information. This distributed network coding for conceptual processes has also been called “experiential brain system” (e.g., Ghio and Tettamanti, 2016).

The idea that language processing activates sensorimotor areas of the brain has been supported by neuroimaging and neuromodulation studies focusing on the processing of nouns, adjectives, verbs and sentences including actions performed by specific body parts or manipulable objects. These studies suggested that primary and secondary motor cortices were regularly involved (Hauk et al., 2004; Buccino et al., 2005; Pulvermüller et al., 2005; Tettamanti et al., 2005; Aziz-Zadeh et al., 2006; Boulenger et al., 2009; Papeo et al., 2009; Alemanno et al., 2012; Gough et al., 2013; Innocenti et al., 2014; Gianelli and Dalla Volta, 2015). Similarly, in studies on emotion, mimetic muscles have been shown to react to emotional words and sentences (Havas et al., 2007; Foroni and Semin, 2009, 2013; Havas et al., 2010; Davis et al., 2015; Foroni, 2015; Fino et al., 2016; Baumeister et al., 2017). Others have also shown correlations between the impairment in action word processing (e.g., “to pour,” “to wave”) and the impairment in action performance, assessed using a visually guided reaching task (e.g., Desai et al., 2015). Finally, some have also shown that a virtual transient lesion induced by repetitive Transcranial Magnetic Stimulation (TMS) or transcranial Direct Current Stimulation (tDCS) over the premotor and motor cortex affects comprehension of action related language (e.g., Willems et al., 2011; Tremblay et al., 2012; Vukovic et al., 2017; Gijssels et al., 2018).

In the clinical setting – following the seminal work by Warrington and McCarthy (1987) – the idea that different “weighting values” from independent perceptual channels could subserve different categories of knowledge is rather undisputed. In their paper, Warrington and McCarthy (1987) presented a severe dysphasic patient who showed impairment in selecting objects (and not food or animate beings) as well as specifically small manipulable objects (and not large man-made objects). Later clinical studies confirmed the interaction between language processing and the activation of perceptuo- and sensori-motor brain areas. For example, Arévalo et al. (2007) showed that manipulable words (e.g., “comb,” “kite”) were distinct to non-manipulable ones (e.g., “smoke,” “moon”), not only behaviorally, but also in their associated activated brain areas. Others have shown that lesions to the sensorimotor areas were associated with impaired processing of lexical and conceptual knowledge of actions (e.g., Kemmerer et al., 2012). In fact, sensorimotor network impairment – due to neurodegenerative diseases – has been shown to selectively compromise the processing of action verbs, motor-language coupling, syntax, and the processing of graspable objects (e.g., Bak et al., 2006; Cotelli et al., 2007; Cardona et al., 2013; Fernandino et al., 2013a,b; Kargieman et al., 2014; Birba et al., 2017; Buccino et al., 2017a; Cotelli et al., 2018). Note that (1) these effects seem to be independent of the general cognitive functioning and of the actual manifestation of the symptoms (e.g., Bocanegra et al., 2015, 2017; García et al., 2017), and that (2) they have not always been found. For example, in some studies, lesions to the motor cortex did not cause deficits in action word processing (e.g., Papeo et al., 2010; Maieron et al., 2013) ^1^. Studies such as these do question the very necessity of activating sensorimotor structures when processing language. They reflect the idea that although embodied cognition is an interesting concept, it is unlikely that *all* our cognition is grounded in sensorimotor experiences (Goldinger et al., 2016). In fact, most contemporary embodied theories do claim that grounded cognition *complements* existing accounts, without the presumption of replacing them, yet it offers new opportunities to study basic cognitive processes (Barsalou, 2016). Hence, despite conceptual controversies (e.g., Mahon and Caramazza, 2008; Papeo et al., 2013; Caramazza et al., 2014; Martin, 2016), the idea that perceptuo- and sensori-motor information is activated when semantic representations are accessed (Meteyard et al., 2012) is extremely interesting in terms of bilingualism and clinical implications. In terms of the former implications, a central issue has been whether lexico-semantic representations are shared or distinct between L2 and L1 (the mother tongue). In terms of the latter, if L2 is less (or not) embodied, clinicians – often confronted to patients whose first language is not the language of rehabilitation – could choose different therapy strategies (more related to action observation or gestures) in L1 but not in L2. We strongly believe that understanding how both languages are represented in the brain and how they interact with one another will help diagnosing and optimizing rehabilitation strategies and health care. To our knowledge, only two other reviews have discussed embodiment and bilingualism: one focusing on emotion studies (Pavlenko, 2012), and one theoretical paper discussing embodiment predictions in bilingualism and presenting clinical implications for children with a Developmental Language Disorder (Adams, 2016). In the present review we wish to further the latter and stress the relevance of studying embodiment in L2 by (1) discussing bilingual language models from this perspective, (2) presenting studies that have linked L2 and embodiment, and (3) calling attention to the concrete clinical implications of the processes at stake.

Note that to keep the focus of the present paper specifically on embodiment and second language lexico-semantic representations and processing (and the subsequent clinical implications), we only briefly mention the work on embodiment *while acquiring* a second language. Although slightly satellite to the present concerns, research on the latter has also raised some important issues for bilingualism research (see for example Macedonia, 2014; Wellsby and Pexman, 2014; Buccino and Mezzadri, 2015; Macedonia and Mueller, 2016)^2^.

## Bilingual Language Models and Embodiment

### The Influence of Proficiency, Immersion and Age of Acquisition on Semantic Representations in Bilingual Models

Current models of bilingualism assume that, when processing a word (either in L1 or L2), after an initial language specific visual processing (Khateb et al., 2016), associated lexico-semantics is activated for both languages (e.g., van Heuven and Dijkstra, 2010; Moon and Jiang, 2012). They also assume that the parallel activation of the two languages is modulated by subject-related factors, such as age of acquisition (AoA; i.e., the age at which bilinguals begin to learn L2, Hernandez and Li, 2007), L2 exposure and/or L2 proficiency. Importantly though, these models do differ in the way they conceptualize lexico-semantic systems. The Revised Hierarchical model (RHM) (Kroll and Stewart, 1994), for example, assumes that each language has a specific lexical system, yet both languages share semantic representations that are stored in a common memory system. In this framework, L2 to L1 connections are more developed than vice-versa, but with increasing proficiency, the strength of L2 connections changes. Other models, such as the Bilingual Interactive Activation Plus model (BIA+) by Dijkstra and van Heuven (2002), assume that the lexical representations of the two languages are somehow integrated. As such, access to the orthographic, phonologic, or semantic representations is non-selective between languages. Dijkstra and van Heuven (2002) and van Heuven and Dijkstra (2010) further discuss how the proficiency of a language relies on the frequency of word usage. As such, it is linked to the rapidity by which those words’ representations are activated. Therefore, in case of low L2 proficiency, the authors argue for a temporal delay to access representations in L2 compared to L1.

In addition to language proficiency, more global exposure to L2 environment plays a role in semantic processing (e.g., Perani et al., 2003; Stein et al., 2014), although these factors are most likely interdependent. Exposure increases proficiency, even to the extent – in extreme cases – of hindering lexical access in L1 (e.g., Linck et al., 2009). Similarly, L2 proficiency is linked to age of acquisition (e.g., Johnson and Newport, 1989). However, L2 proficiency and AoA have been suggested to have different roles in language processing. In particular, language proficiency seems to be more influential than AoA in semantic processes, while AoA would rather play a role in syntactic knowledge (Wartenburger et al., 2003; Abutalebi, 2008). Some have questioned this assumption (e.g., Izura and Ellis, 2004; Isel et al., 2010; Sabourin et al., 2014), suggesting that AoA’s influence was also on the lexico-semantic level. This is in line with the model advanced by Silverberg and Samuel (2004), which postulates a common semantic system between languages only in the case of early AoA. The conceptual environment may be similar, yet only if the two languages are acquired at a similar age. For late L2 learners, the conceptual context has been shaped by years of experiences in L1. This vision is similar in the Sense Model (Finkbeiner et al., 2004), which postulates that L2 lexical semantic representations have less “senses” associated with them in comparison to those in L1.

Semantic representations in bilingual models are therefore differently influenced by proficiency, exposure and age of acquisition, all factors to be taken into account in predicting embodiment in L2.

### Embodiment Predictions for L2 and Their Impact on Language Models

Despite some evidence suggesting sensorimotor involvement in L1 semantic processing, to our knowledge, only few studies have investigated such involvement in L2 processing. The lack of studies on the topic could be explained by two different, yet related assumptions. First, when considering early bilinguals, given that both languages are learnt in the same cultural context^3^, one could assume an overlap of sensorimotor information between the two different languages (Adams, 2016). Second, and contrariwise, in late bilinguals, L2 is often acquired explicitly in a school context, hence without a true involvement of sensory modalities. As such, sensorimotor activation in the two languages should be different, with less rich or direct connections to the sensorimotor cortex for the second language (Perani and Abutalebi, 2005; Pavlenko, 2007; Eilola and Havelka, 2011; Dudschig et al., 2014; see also Declarative/Procedural model on implicit and explicit language learning, Aglioti, 1999; Ullman, 2001, 2004; Paradis, 2004; Hamrick et al., 2018). Yet, if semantic representations are shared between L1 and L2, as assumed by some models of bilingualism, we should not expect a difference in the embodiment of the two languages. One could also argue that in moderately proficient bilinguals (and late AoA), the link between the L2 lexical store and the semantic system is most likely not as developed as that of L1. Consequently, such a weaker connection could translate to different embodiment effects in L2.

Transferring this assumption into clinical predictions, the assessment and rehabilitation of a patient in L2 – acquired late and/or less proficient – could depend on the patient’s embodiment of L1 as well as the possible transfer between languages. It could also depend on the way the two languages are stored. Even if – as assumed by models considering separate stores of concepts for both languages (e.g., Finkbeiner et al., 2004; Silverberg and Samuel, 2004) – the path to access semantic representations is not influenced by a delayed access through L1, the strength of connections between semantics and sensorimotor structures could still vary. Consequently, from a clinical standpoint, both the assessment and the therapy of the lexico-semantic system could be different depending on the language at hand (i.e., L1 or L2). Namely, although specific language tasks may constitute potential markers for movement disorders in L2 – as they do in L1 (e.g., Cardona et al., 2013; Birba et al., 2017; García et al., 2017, 2018) –, this would only be the case if L2 was grounded in the motor system (see section Motor-Language Interactions) and it may depend on its actual degree of embodiment. In the same vein, any transfer of therapy improvement from one language to another is more likely if the same linguistic processes are targeted, such as lexical or phonological encoding (e.g., Laganaro and Overton Venet, 2001). The transfer of outcomes from L1 to L2 would hence be larger if semantic representations are shared, as suggested by some of the bilingual models discussed earlier (e.g., Dijkstra and van Heuven, 2002).

Investigating the sensorimotor activation in L2 – and its therapeutic context – could also offer some insight on models of L1, providing further understanding of the timing of sensorimotor involvement in language processing. Besides this debate (e.g., Mahon and Caramazza, 2008; Postle et al., 2013), answering such a question could generally help us to understand the role of sensorimotor language therapies. We could even argue that a better grasp of the involvement of sensorimotor structures in both L1 and L2 could further models of language representation as well as models of motor-language coupling (e.g., HANDLE, García and Ibáñez, 2016) and of language acquisition (e.g., ABL model of Glenberg and Gallese, 2012). In fact, some language acquisition and development models have already taken embodiment evidence into account. For example, the Word as Social Tool (WAT) model(Borghi and Cimatti, 2009) considers words not only as a referent, but also as a tool to operate in the world. This model already posits different modes of acquisition, namely perceptual, linguistic or mixed, with the level of embodiment depending on these modes (Scorolli et al., 2011). Such a model could be helpful in making predictions for future research in L2 learning and recovery. As an example, while acquiring a language, or in language therapies fostering interactions (e.g., CIAT by Pulvermüller et al., 2001), L2 could be better acquired or retrieved with increasing amounts of social or embodied experiences.

## Studies on L2 Embodied Semantic in Healthy Populations

Although we have, so far, only presented L2 and embodiment as predictions and conjectures, some studies specifically addressing this issue do exist, and we critically discuss them next, raising some of the remaining open questions not yet answered. To facilitate a global perspective on those studies, we present in Tables 1, 2 summaries of their methodological, theoretical and interpretative essence.

**Table 1:** Methodological description of the reviewed studies.

Authors	Type of study	L1	L2	N for analyzed data	Proficiency	Immersion	AOA	Task	Stimuli
Bergen et al., 2010	Behavioral	Experiment 1: L1 English; Experiment 4: L1 various, L2 English	English	39 (experiment 1); 35 (experiment 4)	General: High (students with a min TOEFL score). Task-specific: passive lexical knowledge test	Not reported	Not reported	Forced choice matching task	Verbs (written and imaged)
Buccino et al., 2017b	Behavioral	Italian	English	23	High, C1 (CEFR)	Not reported	Not reported	Go-no go paradigm	Photos and nouns of graspable and non-graspable objects; pseudowords and scrambled images
Dudschig et al., 2014	Behavioral (Experiment 1)	German	English	20	Not reported	Not immersed. Participants never lived in English speaking country	Late bilinguals.11–13 y.o.	Modified Stroop (response to the words’ ink color with an upward or downward arm movement)	Written L1 and L2 words referring to entitieswith a typical location (e.g., star, mole)
	Behavioral (Experiment 2)	German	English	20	Not reported	Not immersed. Participants never lived in English speaking country	Late bilinguals.11–13 y.o.		Written L2 words referring to entitieswith a typical location (e.g., star, mole)
	Behavioral (Experiment 3)	German	English	20	Not reported	Not immersed. Participants never lived in English speaking country	Late bilinguals.11–13y.o.		Written L2 words referring to an emotion (e.g., happy, sad)
Vukovic and Williams, 2014	Behavioral	Dutch	English	20	High (self-reported 6.5 on a 7-point Likert scale)	Varying: *M* = 3.85 y, *SD* = 5.37 y in English speaking country	*M* = 8.65 y *SD* = 3.32 y	Judge whether presented pictures depicted something mentioned in a previously heard sentence	English sentence-picture pairs using interlingual English–Dutch homophones (e.g., “bone,” which in Dutch sounds likethe word “boon” [beans]/bo:n/) occurring in the final position.
Ahlberg et al., 2017	Behavioral	Group 1: German; group 2: Russian and English; group 3: Turkish and Korean	German	Group 1: 47; group2: 45; group 3: 42	From A2 to C2, self-reported CEFR levels	Immersed. All participants were students or employees in Germany	Group 2: 8–28 y.o.; group3: 3–26 y.o.	Modified Stroop (response to the words’ ink color with an upward or downward arm movement)	Spatial prepositions: “auf”, “unter” and “über” and the filler “ab”
Qian, 2016	Behavioral	Chinese	English	30 (15 per group)	High (group A) and low (group B). Group A has passed the test for English Majors Band 4 (TEM-4), while group B has no certificate of College English Test Band-4 (CET-4)	All participants lived in China. No immersion reported. Group A students are majoring in English	Not reported	Semantic judgment	Written high vs. low power nouns in L1 and L2
Sheikh and Titone, 2016	Behavioral (eye-movementmeasures)	French	English	34	Self-reported measures: modified LEAP-Q; Marian et al., 2007, with a 7-point scale from 1 (beginner) to 7 (near-native)	Self-reported measures: modified LEAP-Q; Marian et al., 2007, with a 7-point scale assessing the amount of the L2 use in different contexts, and the percentage of time exposed to L2	Not reported	Natural sentence reading	Semantically neutral sentences in whichembedded L2 target words varied onemotional valence (negative, neutral andpositive), frequency and concreteness
De Grauwe et al., 2014	Neurophysiological (fMRI)	Group 1: Dutch group 2: German	Dutch	Group 1: 20 group 2: 18	High (min. 67.5% accuracy in a online lexical decision task, LexTALE)	At least 1.5 years living in the Netherlands plus regular usage of L2	Late: *M* = 19.94, *SD* = 2.88	Lexical decision task	Written motor- and non-motor cognate and non-cognate verbs/pseudwords
Xue et al., 2015	Neurophysiological (EEG)	Chinese	English	17	High (near-balanced), assessed with a self rating scale, and all passed a national test for English majors, similar to TOEFL	Students majoring in English, but living in China	Not reported	Sentence acceptability judgment	Written high and low BOI words embedded in segmented sentences characterized by rich and poor sensorimotor context
Vukovic and Shtyrov, 2014	Neurophysiological (EEG)	German	English	18	High, measured by self-rated proficiency scores (7-point Likert scale, *M* = 6.10, *SD* = 0.70) and scores on the LexTALE (Lemhöfer and Broersma, 2012) English vocabulary test (*M* = 81.82, *SD* = 22.34)	Not reported. Participants started learning English as part of formal education in Germany	Late (mean 10.19 y.o.,*SD* = 2.19)	Passive reading	Written L1 and L2 action and abstract prime-probe verb pairs
Foroni, 2015	Neurophysiological (EMG)	Dutch	English	26	Good fluency self-reported	Not reported	Late (after 12 y.o.)	Passive exposure to affirmative and negative sentences followed by a simple classification of arrow directions (left orright)	Written L2 affirmative and negative sentences either relevant (e.g., *I am smiling*) or irrelevant (e.g., *I am frowning*) to the target muscle under examination (zygomatic major)
Baumeister et al., 2017	Neurophysiological (EMG)	English or Spanish	Spanish or English	26	Advance level self assessed from 1 to 10 (LEAP-Q; Marian et al., 2007) and letter fluency task	At least 12 months in their L2 speaking country	Not clearly reported. Most of them have a late AoA (started to learn L2 in a classroom setting)	Facial muscle EMG activity and SC responses were obtained during the encoding phase of a classical memory task, in which participants performed a categorization task, which required them to categorize words into “associated to emotion” or “not associated to emotion.”	Written L1 and L2 emotion-laden words


**Table 2:** Theoretical description of the reviewed studies.

	Authors	Aim of the study	Comparison	Embodiment effect	Conclusions on L2 embodiment	L2 vs. L1 embodiment
Behavioral	Bergen et al., 2010	To investigate if (1) action and language understanding use the same motor circuitry and if (2) this motor activation plays a functional role in language understanding	Descriptive comparison between L1 and L2 and between subjects	Slower RTs when the image and verb shared an effector, than when they did not share an effector	• “Like native speakers, these nonnative speakers relied on motor structure activation to understand words” • “Language proficiency (as measured by overall accuracy in the main experiment) correlated positively with effect size”	L2 is embodied/ No statistical comparison with L1
	Buccino et al., 2017b	To test if L2 speakers showed the same kind of modulation of motor responses as participants in a previous experiment (Marino et al., 2014)	Descriptive comparison between L1 and L2 and between subjects (previous study Marino et al., 2014)	Slower RTs during the processing of graspable items as compared to non-graspable ones	• “Fluent speakers of English as L2, showed the same kind of modulation of motor responses as participants in a previous experiment (Marino et al., 2014), where the same kind of stimuli were presented in their L1”	L2 is embodied/ No statistical comparison with L1
	Dudschig et al., 2014	To investigate basic associations between L2 and the sensorimotor system	Statistical comparison between L1 and L2 within subjects	Compatibility effect, reflected in an interaction between the implicit location of words and the direction of the response movement	• “L2 automatically activated motor responses similar to L1 even when L2 was acquired rather late in life (age > 11)” • “Reactivation of experiential traces is not limited to L1 processing”	No difference between L2 and L1 embodiment
			None	Compatibility effect, reflected in an interaction between the implicit location of words and the direction of the response movement		L2 is embodied/ No statistical comparison with L1
			None	Compatibility effect, reflected in an interaction between emotion words (e.g., happy, sad) and the direction of the response movement		L2 is embodied/ No statistical comparison with L1
	Vukovic and Williams, 2014	To investigate the automaticity of embodied simulatory processes in language comprehension, exploiting the fact that both languages are activated during comprehension in bilinguals	None	Slower RTs to target stimuli in the homophone condition, suggesting activation of both languages which, in turn, led to greater competition between alternative meanings	• “Perceptual simulation of L1 meaning occurs during L2 sentence processing”	L2 is embodied/ No statistical comparison with L1
	Ahlberg et al., 2017	To compare embodiment effects related to the processingof spatial prepositions in German native speakers with the embodiment effectspotentially observed in different groups of L2 learners	Statistical comparison within language (German as L1 and as L2) and between subjects	Compatibility effect, reflected in an interaction between the meaning of the spatial prepositions and the direction of the response movement	• “Reactivation of experiential traces plays an important role not only in L1 processing but also in the processing of L2, even if this *[L2]* is quite dissimilar to the participants’ native language”… “We found clear processing differences between this group *[dissimilar-L1 group]* and the other two groups” • “We found [...] also clear differences between speakers of different L1s” + “L2 language proficiency modulate the embodiment effect”	L2 is embodied, but differently from L1
	Qian, 2016	Investigate if (1) Chinese English learners with different proficiency levels activate vertical spatial metaphors in processing power words, and if (2) L2 proficiency has an impact on the mental representations in processing power words	Statistical comparison between L1 and L2 within participants, and statistical comparison within L2 between participants (high vs. low proficient)	Faster RTs for power words presented in the upper vs. lower part of the screen	• “Chinese English learners tend to show stronger mental representation abilities to their first language than their second language” • “High-level second language learners have stronger representation ability than low-level learners in understanding English words of power” • “For RT, there’s an interaction between power words and L2 proficiency. [..] Learners’ second language proficiency had an impact on their mental representation ability.”	L2 is embodied, but differently from L1
	Sheikh and Titone, 2016	To investigate if bilingual readers (1) exhibit L2 emotional word processing effects, (2) show facilitation of negative words byconcreteness, like neutral words, and if (3) differences in L2 proficiency predict facilitationby concreteness and frequency, but not emotionality	Statistical comparison within L2 across participants with varying L2 proficiency and statistical comparison within language (English as L1 and as L2) between participants (previous study Sheikh and Titone, 2013)	Shorter gaze duration (concreteness facilitation) for negative and neutral low-frequency words, but only at high levels of proficiency	• “Previous work on L1 embodiment indicates that a concreteness advantage, where observed, is diagnostic of emotional neutrality because it does not occur for emotionally charged words (Sheikh and Titone, 2013).” • “Bilinguals have emotionally disembodied negative words during L2 reading, and that these words are instead grounded in sensorimotor experiences like neutral words.” • “L2 proficiency predicts concreteness advantages but not emotional advantages during natural reading.”	L2 is embodied, but differently from L1
Neurophysiological	De Grauwe et al., 2014	(Among others) to investigate if L2 speakers show embodiment effects like L1 speakers	Statistical comparison between Dutch as L1 and Dutch as L2 between participants	Motor verbs yielded significantly higher activation in sensorimotor ROIs than non-motor verbs	• “Both groups *[L1 and L2 speakers]* displayed higher activation for motor than for non-motor verbs in motor and somatosensory brain areas. These activations were evident with both cognate and non-cognate verbs, indicating that they were not due to transfer from the native language of the L2 speakers.”	No difference between L2 and L1 embodiment
	Xue et al., 2015	To investigate if (1) richer sensorimotor context lead to increased sensitivity to the anticipated sensorimotor consequences of BOI effect, and if (2) the context and use of BOI word processing activate sensory-and motor-related brain areas	None	Sensorimotor context had an impact on the processing of BOI words that this modulation did activate related sensorimotor areas	• “Action- and perception-related brain areas for L2 words are activated, indicating that the semantic representations for L2 learners are rich enough for the sensorimotor-related activation.”	L2 is embodied/No comparison with L1
	Vukovic and Shtyrov, 2014	To investigate if the motor cortex of bilingual subjects shows differential involvement in processingaction semantics of native and non-native words	Statistical comparison between L1 and L2 within participants	Action related words elicited increased motor brain activity reflected in stronger ERD	• “Processing of action verbs was accompanied byearly motor activation for probe stimuli in both languages of bilingual subjects, as reflected in desynchronisation of the EEG mu rhythm. Furthermore, at the level of sensor data and source activation clusters, we observed that this motor activation is stronger in the L1, likely due to highly integrated action–perception circuits formed as a result of rich linguistic experience.” • At the sensor level, L1 probes elicited a larger desynchronization in the right-hemisphere cluster than L2 probes did; at the source level, L1 action primes, unlike L2 ones, produce more ERD.	L2 is embodied, but differently from L1
	Foroni, 2015	To examine if the processing of affirmativeand negative sentences in L2 relies on the same somatic bases as that of L1 (Foroni and Semin, 2009, 2013)	Statistical comparison between L1 and L2 between participants (across studies, L1 data from Foroni and Semin, 2013)	When participants readaffirmative sentences (e.g., ‘I am smiling’) the relevant muscle (i.e., zygomatic major) activates; however, when they read negativesentences (e.g., ‘I am not smiling’), the relevant muscleis inhibited only in L1	• “The magnitude of the somatic reaction in L2 is smaller than the one reported for L1” • “The weaker magnitude of the somatic simulation for L2 compared to the one reported for L1 is generally in line with the argument that the different socialization histories of L1 and L2 are reflected in different degree of embodiment” • For affirmative relevant sentences: L1 shows larger activation compared to L2 at 600 and 1000 ms period with the 800ms period showing a marginal significant difference. For negative relevant sentences, significant relaxation/inhibition in L1 and no effect in L2.	L2 is embodied, but differently from L1
	Baumeister et al., 2017	To investigate the link between embodied processes and memory for emotional content within the frame of L1 and L2 processing	Statistical comparison between L1 and L2 within participants	Differential activation in the zygomaticus and corrugator muscle in response to happy vs. angry words	• “The overall results of the EMG and SC recordings suggested some reductions and differences in embodied simulations of emotional L2 words in comparison with emotional L1 words” • “The difference between L1 and L2 processing became particularly clear in the corrugator muscle, which showed typical response patterns to emotional L1 words but no detectable responses to emotional L2 words.” “The present results complement and extend Foroni’s results showing a significant difference for the corrugator muscle and, thus, together they support an embodiment account of emotion processing and a reduced embodiment in L2.” • Result indicates a later onset and shorter duration of specifiable zygomaticus activity in response to happy vs. angry words in L2 compared with L1	L2 is embodied, but differently from L2


### Behavioral Studies

Bergen et al. (2010) assessed sensorimotor activation when native (Experiment 1) and non-native English speakers (Experiment 4) process words in English. In their task, participants had to indicate if a written verb was or was not a good description for an action depicted in a preceding image. The verbs could either match the image (e.g., an image of someone running with the verb *run*) or mismatch it, yet refer to actions using the same (e.g., kick) or a different effector (e.g., drink). In the mismatch condition, participants were slower to correctly respond when the verb used the same effector than when it was different. This interference effect was similar for non-native and native English speakers, suggesting that both groups relied on sensorimotor activation to understand verbs. Still, English proficiency, calculated as accuracy in the task, was positively correlated with the size of the effect (Bergen et al., 2010).

In a similar vein, in Buccino et al. (2017b), Italian students performed a *go–no go* task in which English nouns and pictures of graspable and non-graspable objects were shown. The stimuli either referred to real objects (i.e., *go* condition) or to meaningless ones (i.e., pseudo-words and scrambled images; *no-go* condition). In the *go* condition, participants responded significantly slower when nouns and pictures of graspable objects were presented. According to the authors, activating the motor system both when manually responding and when processing a graspable object comes with a cognitive cost, hence the slower response times. A similar effect was found in a previous study by Marino et al. (2014), who tested English native speakers, leading Buccino et al. (2017b) to conclude that motor response modulation was similar in L1 and in L2.

Dudschig et al. (2014) tested a similar effect in L1-German L2-English late bilinguals. In their adapted Stroop task, participants had to identify colors of the presented words using downward or upward motor responses. The presented words referred to entities with a typical location (e.g., *star*, *root*) (Experiments 1, 2) or emotions (Experiment 3). The authors showed that responses were faster when words matched participants’ motor responses (e.g., upward response with the word *star* or the word *happy*, experientially associated with “up”) in both languages.

According to Dudschig et al. (2014), such facilitation could be due to (a) an automatic activation of L1 words and their experiential associations when processing L2 words or (b) a direct connection made during L2 learning to the sensorimotor experiences made during L1 learning. Even if the latter interpretation was favored due to the early onset of the embodiment effect, the former cannot be excluded, as the results by Vukovic and Williams (2014) suggest. In their study, 24 L1-Dutch L2-English bilinguals listened to English sentences implying physical distances (e.g., *On the plate in front of you, you can see a bone* vs. *On the plate at the far end of the table, you can see a bone*), with interlingual homophones (e.g., “bone,” which in Dutch sounds like the word “boon” [beans]/bo:n/). After each sentence, a picture of the target object was presented to participants, in small or large dimensions. Large pictures were congruent to the sentences implying near distances and the small ones to those implying far distances. Participants were slower in judging if an object had been mentioned in the sentence previously heard if that object was a homophone in L1 with perceptual features congruent to the distance implied by the sentence. The authors argued that a perceptual simulation supports an early and parallel semantic processing in the two languages. Namely, bilinguals mentally simulate detailed perceptual features of L1 homophones while processing L2.

In their adapted Stroop task, Ahlberg et al. (2017) used the German spatial prepositions *auf* [on], *über* [above] and *unter* [under/below]. Participants, native or non-native German speakers – one non-native group with a similar use of spatial prepositions (i.e., English or Russian) and one non-native group with a dissimilar use of spatial prepositions (i.e., Turkish or Korean) – had to identify colors of the presented words using an upward or a downward hand movement. Results showed a different pattern of embodiment depending on L2-proficiency and on the corresponding use of the prepositions in the non-native groups’ L1. However, all three groups (native, non-native similar and non-native dissimilar) were similarly affected by the Stroop task: responses were faster when the hand movement matched the spatial direction of the preposition. The authors concluded that processing a word in L2 does activate an experiential trace created in L1. This in turn corresponds to the first interpretation of Dudschig et al. (2014) and is in line with the results of Vukovic and Williams (2014), supporting the idea of a co-activation of L1 and L2. However, it should also be noted that a co-activation of L1 and L2 does not necessarily rule out the possibility of a direct, newly built connection between L2 words and the experiential representations.

Others have been less inclined to suggest that L2 was embodied, at least as strongly as L1. For example, Qian (2016) showed stronger embodiment effects in L1 than in L2. In her paper, she investigated the way the vertical spatial metaphor of the concept of “power” was processed in L1-Chinese L2-English speakers, half of them having high L2-English proficiency. Participants had to judge if the nouns presented on the upper or lower part of the screen were related to “power” or not. Words associated with higher power were facilitated when presented in the upper part of the screen, whereas words associated with lower power were facilitated when presented in the lower part of the screen. This effect was, however, stronger in L1 than in L2, and was stronger in L2 for higher proficient L2 speakers. Note that some limitations of this study, both methodological and statistical (e.g., lack of detailed report) force us to consider its results with caution.

Still, a number of language studies, in which emotional valence of the stimuli was manipulated, have also observed differences in L1 and L2 affective processing, suggesting that the languages may be embodied to a different extent, especially in the case of late acquired L2 (Pavlenko, 2012). For example, Sheikh and Titone (2016), focusing on early stages of lexical processing, found L1-French L2-English speakers to be faster to process positive words than neutral words (first time reading passes), but not faster to read negative words than neutral ones. This was not the case in their previous work on L1 (Sheikh and Titone, 2013), suggesting, as raised by the authors, that negative words do not seem to be grounded in emotional experiences in L2. However, the concreteness advantage (sensorimotor grounding) in L1 was present for low frequent neutral words but not for emotional words (Sheikh and Titone, 2013), while in L2 it was present for both neutral and negative high frequent words (Sheikh and Titone, 2016). Moreover, results showed that L2 proficiency positively correlated with the concreteness advantage.

In sum, behavioral studies revealed that L2 is very likely embodied. Firm conclusions regarding the degree to which L2 is embodied remains to be clarified, as some studies report differences in L1 vs. L2 embodiment (Qian, 2016; Sheikh and Titone, 2016; Ahlberg et al., 2017) whilst others did not find such differences (Dudschig et al., 2014, Experiment 1), or did not perform direct statistical comparisons between languages (Bergen et al., 2010; Vukovic and Williams, 2014; Buccino et al., 2017b). In Tables 1, 2, we summarize the studies that have investigated these issues.

### (Neuro-)Physiological Studies

To our knowledge, De Grauwe et al. (2014) were the first to conduct an fMRI study to investigate embodiment in L2. In a lexical decision task, highly proficient L1-German L2-Dutch and Dutch native speakers were presented with motor and non-motor cognate or non-cognate^4^ verbs in Dutch. Results showed a significantly stronger activation in motor and somatosensory areas for motor verbs, regardless of the cognate status of the verbs. This was the case for both language groups. De Grauwe and colleagues consequently suggested L2 representations to be rich enough to activate similar motor-related areas as L1. Note that as all participants were late highly proficient bilinguals, the impact of proficiency and AoA on the embodiment effect cannot be established beyond conjecture (De Grauwe et al., 2014).

In a similar vein, Xue et al. (2015) presented L1-Chinese L2-English participants with high (e.g., *crumb*) and low (e.g., *lace*) body-object interaction (BOI) English words. These words were imbedded in high (e.g., *you brush the small sticky* *crumb*) and low (e.g., *you wear a string of cotton lace*) sensorimotor contexts. Highly proficient L2-English participants judged sentence acceptability while ERPs time-locked to the onset of the high vs. low BOI words in rich and poor context were recorded. The results showed a marginal sensorimotor context effect reflected in ERP differences in both the P2 and N400 components. The authors suggested that this effect was related to differential activation of sensorimotor areas, based on observed differences in electrodes over the sensorimotor cortex.

Other studies including neurophysiological measures have also supported the notion that bilinguals’ L2 is less embodied than L1. Vukovic and Shtyrov (2014), for example, examined mu-rhythm event-related desynchronization as an index of motor cortex activity in response to L1 and L2 abstract and action prime-probe verb pairs. Highly proficient L1-German L2-English speakers performed a passive reading task while an electroencephalogram was recorded. Analysis of motor-related EEG oscillations revealed that cortical motor activation was present in both L1 and L2 around 150 ms post-stimulus. Yet, L1 probe verbs elicited stronger sensorimotor brain activation than L2 probes. Foroni (2015) measured the strength of zygomatic muscle activation when participants read relevant (i.e., to the zygomatic muscle) affirmative and negative short sentences (e.g., *I am…* or *I am not… smiling*) and irrelevant ones (e.g., *I am…* or *I am not frowning*). Having *negative* sentences provided the authors with an alleged muscle relaxation condition, offering a way to further evaluate inhibition processes. Interestingly, the results showed stronger activation of the zygomatic muscle when participants read affirmative sentences, mimicking the results found in L1 (Foroni and Semin, 2013). Yet, the magnitude of the somatic activation was smaller in L2 than L1. Moreover, differently from L1 (Foroni and Semin, 2013), there was no relaxation of the relevant muscles when participants read negative sentences in L2. Therefore, embodiment in L2 was only partial.

These results are corroborated by those of Baumeister et al. (2017) on emotion and memory. Grounded in the idea that emotional words are better remembered than neutral ones, they recorded electromyography and skin conductance of 26 late L1-Spanish L2-English bilinguals during a categorization task of emotional and neutral words in both L1 and L2. A day later, participants went through a memory recognition task. Although their results were not decisive (i.e., marginally significant), there were some trends indicating that (a) there was a reduced, delayed and short-lived motor resonance in response to emotional words in L2, and that (b) a strong motor resonance would lead to better memorizing of emotional words.

Some studies on bilingualism and emotions (e.g., Harris et al., 2003; Harris, 2004; Caldwell-Harris, 2015; Hsu et al., 2015) have also suggested that L2 emotional words evoke less autonomic physiological response than L1 words, leading some authors to describe L2 as “disembodied” (for a review see Pavlenko, 2012, 2017). However, as Sheikh and Titone (2013) have pointed out, there might be a difference between *emotionally grounded* and *sensorimotor grounded* concepts, difference which goes beyond the scope of this paper.

In sum, (neuro-)physiological data globally confirm findings from behavioral ones on L2 embodiment, independent of the techniques used. Some issues still remain unanswered though, especially those pertaining to the degree by which L2 is embodied and to the roles of AoA, proficiency and immersion (see Tables 1, 2 for a summary of these studies).

### Critical Synthesis

The role of the sensorimotor system in L2 language processing has not received much attention, yet we have tried to gather and collate the few studies specifically focused on this issue. Crucially, all these studies show an embodiment effect during the lexico-semantic processing of L2 (see Table 2), independently of the techniques used (behavioral or neurophysiological) or of the specific aim of the study in question.

Interestingly, eight out of the twelve studies reported in this review *statistically* compared the degree of L2 vs. L1 embodiment (see Tables 1, 2 for a summary), and only two of them concluded a similar embodiment for both languages (De Grauwe et al., 2014; Dudschig et al., 2014). However, in the latter two studies, the extent of true similarity would need further investigation. For example, Dudschig et al. (2014) reported a slightly stronger significance of embodiment effect in L1 vs. L2, without delving into it in the discussion, and De Grauwe et al. (2014) found different patterns in sensorimotor activation between L1 and L2, which they explained in terms of methodological parameters. All the other studies discussed in this review report that L2 is differently embodied than L1, usually expressed as a *lower degree* (Vukovic and Shtyrov, 2014; Foroni, 2015; Qian, 2016; Baumeister et al., 2017) of embodiment in L2 or as a *different pattern* (Sheikh and Titone, 2016; Ahlberg et al., 2017) of embodiment. Such a difference may be explained by different factors discussed hereafter.

Several studies suggest an influence of participants’ L2 proficiency on the degree of L2 embodiment. In terms of the RHM model (Kroll and Stewart, 1994), and as suggested by others (e.g., Qian, 2016), this could be explained by an asymmetry in the strength of the connections between words and their representations in the two languages, mainly characterized by stronger links, and hence faster access to meaning, in L1. In contrast, access to L2 representations would require mediation via L1, especially in case of low L2 proficiency. This entails a later sensorimotor involvement when L2 proficiency is low compared to when it is high, or compared to L1. Such differences in the degree of L2 embodiment would also be in line with the BIA+ model (Dijkstra and van Heuven, 2002) assuming later semantic access when L2 proficiency is low. However, none of the studies presented can actually reach a definite conclusion as to the role of proficiency, and this for three main reasons. First, L2 proficiency was not always thoroughly assessed, if assessed at all. To provide us with relevant insight into the issues discussed so far, we believe that L2 proficiency should always be assessed, whether it be on objective measures such as receptive (e.g., DIALANG, Zhang and Thompson, 2004), and productive vocabulary (e.g., Productive Vocabulary Levels Test,Laufer and Nation, 1999), and/or subjective ratings from questionnaires including self-evaluation and language background (e.g., LEAPQ, Marian et al., 2007). Second, L2 proficiency was never actually specifically manipulated (except in Qian, 2016, without thorough proficiency assessment). Third and finally, participants’ L2 general proficiency could not always be reflected in the actual lexico-semantical knowledge of the stimuli in the experiment, therefore raising the need to add task-specific measurements of proficiency, as was done by Bergen et al. (2010), who administered a passive lexical knowledge test.

One could further argue that even if proficiency was to be carefully assessed, any embodiment effect could also be accounted for by factors such as exposure to L2 and/or AoA. If the degree of embodiment of L2 depends on the degree to which L1 and L2 share their semantic representations, some models (e.g., Silverberg and Samuel, 2004) would actually assume a common semantic system between languages only in the case of early AoA. Therefore, L2 lexico-semantic processing would involve sensorimotor areas to the same degree as L1 lexico-semantic processing only in case of an early acquired L2. Exposure and AoA have never been manipulated in bilingual studies on embodiment, allegedly the former because it may be highly interrelated to proficiency and the latter because it is usually considered to be less associated with semantic processing. This is rather unfortunate, as representations have been shown to be modulated by exposure when proficiency was kept constant (e.g., Perani et al., 2003), even after a short period (e.g., Dahl and Vulchanova, 2014). Not considering AoA may also be problematic, as AoA could show different effects depending on the nature of L2 learning. Namely, early L2 AoA has been associated with implicit L2 learning, which takes place in a naturalistic setting via sensorimotor experiences, while late L2 AoA has been associated with explicit learning, taking place in the setting of a traditional classroom via amodal instructions. Some studies contrasting different types of L2 learning have been mainly interested in learning and memory performances (e.g., Zimmer, 2001; Repetto et al., 2017; García-Gámez and Macizo, 2018). Other studies have tried to untie the type of learning from AoA. For example, independent of the learning setting, structural changes have been observed in the left inferior parietal cortex, and differences in these changes have been attributed to AoA (Stein et al., 2014).

In fact, the importance of the type of learning for L2 embodiment may be illustrated by studies which show a rapid association between motor areas’ activation, or excitability, and novel labels attributed to actions or tools (e.g., Liuzzi et al., 2010; Fargier et al., 2012; Branscheidt et al., 2017a; Bechtold et al., 2018 in elderly). These studies showed embodiment effects in newly formed L2-like representations, also when experiential traces were not transferred from L1 to L2 (Fargier et al., 2012; Öttl et al., 2017; Bechtold et al., 2018). As such, these studies document the influence of exposure, AoA, and type of learning on grounding language in bodily experiences. Interestingly, and future research on these effects taking a lifespan perspective should consider this, language-induced motor activity in the brain has been shown to change with training (Fargier et al., 2012), and seems to be different between children and adult (Dekker et al., 2014), yet already present in young children (e.g., James and Swain, 2011; see also Inkster et al., 2016). These issues have been well documented (e.g., Macedonia, 2014; Wellsby and Pexman, 2014; Macedonia and Mueller, 2016).

Another factor that could account for differences between L1 vs. L2 embodiment is the linguistic distance between languages, which refers to the extent of similarity between the languages and which has previously been shown to play a role in bilingual language processing (e.g., Lindgren and Muñoz, 2013; Abutalebi et al., 2015; Ghazi-Saidi and Ansaldo, 2017). This factor is usually studied in relation to the ease of learning a second language (e.g., Butler, 2012), or in relation to the phonology and morpho-syntax of languages (e.g., Llama et al., 2010; Zawiszewski et al., 2011). Studies on the influence of linguistic distance on embodiment remain scarce and languages have not been always chosen in a systematic way. For example, some have compared embodiment in languages that are both Germanic (Vukovic and Shtyrov, 2014; Foroni, 2015), others compared a Germanic language to an Italic one (Sheikh and Titone, 2016; Baumeister et al., 2017), and Qian (2016) compared two different language families (i.e., Sino-Tibetan and Indo-European). Essentially, linguistic distance could act as a catalyst for embodiment similarity between L1 and L2. To the best of our knowledge, only one study addressed this issue (i.e., Ahlberg et al., 2017), and found little effect of linguistic distance. In a nutshell, Ahlberg et al. (2017) found similar embodiment effects in L2 (German) for two non-native groups, irrelevant of the linguistic distance between L1 and L2 (i.e., whether or not L1 linguistic properties could easily match to L2). Clearly, more research needs to be carried out to reach definite conclusions.

This issue is nonetheless relevant, especially in studies that (a) involve words with a special status (e.g., cognates, as in De Grauwe et al., 2014; or false friends, as in Degani et al., 2018, and Persici et al., 2019), (b) involve manipulating linguistic properties that differ across languages (e.g., the meaning of spatial prepositions, as in Ahlberg et al., 2017; or the perspective implied by the use of personal pronouns, as in Papeo et al., 2011) or (c) involve an experimental design in which the two languages are intermixed in the same block event (e.g., semantic priming driven by phonological properties, as in Vukovic and Williams, 2014; Degani et al., 2018).

Others have stressed the timing of the motor system involvement as an explanatory factor for the difference between L1 and L2 embodiment. Differences both in the onset of the motor resonance and its duration have been reported by Foroni (2015) and Baumeister et al. (2017). Specifically, their experiments showed that L2 motor resonance had a later onset and shorter duration compared to L1. Latency shifts have previously been associated with delayed lexico-semantic processing for L2 compared to L1 in several neurophysiological studies (e.g., Moreno and Kutas, 2005; Leonard et al., 2010; Newman et al., 2011), in line with the bilingual language models suggesting faster access to meaning in L1, as discussed earlier.

Arguably, these potential explanatory factors – all legitimate – raise an important issue, as to the stages of cognitive processing under investigation. Accordingly, any endeavor to investigate embodiment in L2 should always be very explicit as to which stage of processing is under investigation. This is crucial, as the majority of the studies on this topic used tasks which allegedly access early stages of lexical processing (e.g., a Stroop task or a lexical decision task, where the access to meaning is not necessary; Dudschig et al., 2014; Ahlberg et al., 2017), while others used tasks which require deep semantic processing (e.g., a semantic judgment or a picture-word matching task; e.g., Vukovic and Williams, 2014; Xue et al., 2015; Qian, 2016). As differences in embodiment related to the depth of semantic processing have been shown in L1 (e.g., Willems et al., 2009; Vukovic et al., 2017), we would further argue the motor circuit recruitment to be different between L1 and L2 depending on the task used – consequently the stage of processing accessed – in the experiment.

Importantly, all explanatory factors – to differences between L1 and L2 embodiment – presented so far have been based on studies on *language-to-motor* effects. A more complete (or even different) picture of the interaction between the sensorimotor system and lexico-semantic processing may stem from also examining *motor-to-language* effects. This may be crucial, as we do know, from studies on monolinguals, that experimental manipulations of the sensorimotor system can affect lexico-semantic processing. Sensorimotor system manipulations have been as diverse as motor training (e.g., in healthy Glenberg et al., 2008; Locatelli et al., 2012; in experts Beilock et al., 2008; or with dyslexic children Trevisan et al., 2017), motor limitation (e.g., Bidet-Ildei et al., 2017), or motor brain area stimulation (Willems et al., 2011; Tremblay et al., 2012; Vukovic et al., 2017; Gijssels et al., 2018). To the best of our knowledge, no study has directly assessed *motor-to-language* effects in healthy bilinguals, linking the sensorimotor system and lexico-semantic processing. Interventions on the motor system may help language processing, as much as language-based interventions may contribute to motor improvements, both in L2 and L1. More generally, and this is the focus of the next section, we believe that studies on L2 embodiment may serve also clinical purposes, although this has been only rarely recognized.

## Studies on L2 Embodiment Serving Clinical Purposes

No clinical study has apparently explicitly linked the sensorimotor system to L2 lexico-semantic processing. Nonetheless, some studies on bilingual patients with motor impairment did explore motor-language interactions, yet with somehow different purposes (e.g., syntactic impairment). In the next section, we discuss these studies and corollary hypotheses related to lexico-semantic processing. In the following section, we present some clinical rehabilitation studies – in L1 – that could be interpreted in terms of embodiment (e.g., language-action therapies in aphasic patients) and then extend the discussion to L2, and bilingual rehabilitation outcomes.

### Motor-Language Interactions

Clinical studies on the interaction between motor and L2 language systems have been scarce, yet could document the modulation of motor impairment on L2 processing as well as the impact of L2 impairment on sensorimotor systems.

In Section “Embodiment Predictions for L2 and Their Impact on Language Models” we discussed the idea that L2 lexico-semantic representations should be less grounded in the sensorimotor system – the motor cortex – if L2 is acquired through late explicit learning. This is reminiscent of the Procedural/Declarative model of language acquisition (Ullman, 2001), which distinguishes between procedural memory – which underlies implicit linguistic competences – and declarative memory – which underlies explicit linguistic competences –. The former is implemented in fronto-basal ganglia circuits, whilst the latter is implemented in bilateral medial and temporoparietal structures. In light of this model, Zanini et al. (2004, 2010) and Johari et al. (2013), for example, discussed how implicit grammatical language processing in L1 is more impaired than explicit grammatical language processing in a late L2 in Parkinson’s disease, as one would expect from a disease characterized by an impairment in the fronto-basal ganglia loops. In Johari et al. (2013), Parkinsonian patients did more error in L1 (implicit learning) than in L2 (explicit learning) in all the three administered syntactic tests from the Bilingual Aphasia Test, whilst this was the case only in one subtest for healthy controls. Importantly, these deficits were not correlated to other cognitive measures such as the Mini Mental State Examination, the Wisconsin Card Sorting Test and the Colored Raven Progressive Matrices, illustrating their specific linguistic focus. Similarly, Zanini et al.’s (2004, 2010) Parkinsonian patients showed deficits in syntactic processing and more phonological and morpho-syntactic errors in L1 than in L2, whilst healthy controls had fewer errors in L1 than in L2.

Whilst proficiency, exposure to L2 and AoA were not always carefully considered in studies on healthy participants, these factors were more thoroughly reported in Zanini et al. (2004, 2010) and Johari et al. (2013). In fact, in these studies, both healthy controls and patients (a) were proficient in L2 (based on the number of years and the context of usage), (b) were exposed to L2 on a daily basis, and (c) had acquired L2 late (at 6 years old at school). Participants in Johari et al. (2013) were highly proficient L2 speakers, and L2 was also their dominant language (used every day). Even if not specifically manipulated or formally assessed, Johari and colleagues argued that high L2 proficiency could explain worse performance in L2 in patients vs. controls, whilst the performance in L2 was not affected in lower proficient speakers in Zanini et al. (2004, 2010). The authors suggested that in case of higher proficiency, L2 is more likely to be processed partly implicitly, as L1, hence relying on procedural as well as declarative memory (Hamrick et al., 2018). Clinical studies specifically focusing on L2 lexico-semantic and sensorimotor systems (and their related brain areas) are needed to better understand procedural and declarative language influences on the motor network (and vice-versa). In fact, studies on monolingual patients showed that semantic deficits (declarative knowledge) affect more severely action-related than non-action-related stimuli in Parkinson’s disease (e.g., Cardona et al., 2013; Bocanegra et al., 2015; Gallese and Cuccio, 2018), which does not seem to be predicted by the Procedural/Declarative model (see also Druks and Weekes, 2013). Note that Zanini et al. (2010) did suggest grammatical properties to be accessed during lexical retrieval, and therefore hinting at the idea that lexico-semantic knowledge may be connected to morpho-syntactic properties of language. As such, disentangling syntactic from lexico-semantic processes might not always be possible (e.g., Zwaan et al., 2010; Sell and Kaschak, 2011; Ahlberg et al., 2017).

Data on bilingual Parkinsonian patients also illustrate the Disrupted Motor Grounding Hypothesis (DMGH; Birba et al., 2017), based on neural reuse theories (neural exploitation hypothesis, Gallese and Lakoff, 2005; Gallese, 2008; shared circuit model, Hurley and Chater, 2005; Hurley, 2008; neuronal recycling hypothesis, Dehaene and Cohen, 2007; massive redeployment hypothesis, Anderson, 2007a,b, see Anderson, 2010 for a review). These suggest that low-level neural circuits can be exploited, recycled, and redeployed for other cognitive functions than their original ones. Based on this idea, the DMGH suggests that impairment in the network responsible for sequencing motor information can disrupt the functionally corresponding higher-level mechanism of sequencing words (i.e., syntactic processing).

Importantly, and central to the present paper, the DMGH also predicts lexico-semantic deficits in motor-related disorders. According to the DMGH, action-related meanings, in a somatotopic manner, are mapped onto motor circuits. Accordingly, semantically processing action words and sentences, as well as integrating verbal and motor information, should also be impaired in Parkinsonian patients, which seems to be the case (Boulenger et al., 2008; Cardona et al., 2013; Fernandino et al., 2013a; García and Ibáñez, 2014; Bocanegra et al., 2015; García et al., 2016; Buccino et al., 2017a; Gallese and Cuccio, 2018; see also Bak, 2013 for a review including other motor neuron diseases). For example, in Boulenger et al. (2008), masked priming effects for action words were almost absent in Parkinsonian patients deprived of dopaminergic treatment, whilst they were present – as healthy controls – when they were on Levodopa. The author concluded that their results constituted compelling evidence that lexico-semantic processing depended on the integrity of the motor system (brought by the medication for Parkinsonian patients). Noteworthy, all patients in the studies of Zanini et al. (2004, 2010) and Johari et al. (2013) were on Levodopa or other dopaminergic drugs, but this condition was not enough to restore the intrinsic impairment in syntactic processing. As pointed out by Boulenger et al. (2008) reaction times or error rates for action verbs in their study were not differently affected by the motor impairment or by the dopaminergic treatment. Whether lexico-semantic impairment of action-related meanings and of other verbal and motor information integration is expected in L2 is yet to be examined. At least in L1 patients with basal ganglia impairment, who typically show frontostriatal atrophy, difficulties in motion-related verbal expressions seem to be detectable before the appearance of clinical symptoms (Birba et al., 2017). As such, linguistic diagnostic tasks may help identify Parkinson patients well before the clinical manifestation of the disease (Cardona et al., 2013; García and Ibáñez, 2014; García et al., 2017, 2018). These tasks may also help to identify and stage pre-symptomatic Huntington disease patients (Kargieman et al., 2014).

Questions remain as to the use of linguistic diagnostic tasks in L2. At this point, there is no data to evaluate patients’ sensitivity to L2 tasks that evaluate the processing ease of motion-related verbal expressions. Depending on the grounding of L2, a simple use of a diagnostic L1 task (yet to be generated) may not be adequate. Factors such as AoA and language competence may be critical, together with the presence of emotionally charged content, which might be perceived very differently depending on the language in use (i.e., L1 or L2, see Sheikh and Titone, 2016). Still, the few studies with bilingual Parkinsonian patients suggest that L2 linguistic diagnostic tasks could mimic L1 tasks, even for distant languages. Similar patterns of impairment in each language have been found in speakers of distant languages (e.g., two Indo-European languages in Zanini et al., 2010, and one Indo-European L1 and the other Altaic-Turkic L2 language in Johari et al., 2013). As previously suggested, the extent of language distance and its impact on these issues are yet to be thoroughly examined.

In sum, actual evidence on *motor-to-language* oriented clinical studies show four important findings. First, motor impairments impact lexico-semantic processing of motor related stimuli in L1 (e.g., Bak, 2013; Cardona et al., 2013; Fernandino et al., 2013a; Bocanegra et al., 2015). Second, motor impairments may impact morpho-syntactic processing in L2 (Zanini et al., 2004, 2010; Johari et al., 2013). Third, motor-related interventions could modulate language performances (Boulenger et al., 2008). Fourth and finally, all the factors discussed in the previous sections of this paper (i.e., proficiency, AoA, exposure, distance between languages, type of exposure) may influence the degree of language impairment due to motor-related diseases (Johari et al., 2013).

Although *motor-to-language* clinical studies in L2 may be scarce, there seems to be none on *language-to-motor* effects in L2. In other words, the impact of L2 lexico-semantic processing on motor system has yet to be examined in brain-damaged populations. In monolinguals, some studies did look at the co-occurrence of language and motor impairment in developmental disorders (e.g., Hill, 2001; Sanjeevan et al., 2015) or brain-damaged patients (Desai et al., 2015; for a review see Anderlini et al., 2019).

We believe that, however, weak the *language-to-motor* effects might be in L2 and unhealthy populations, they deserve some empirical attention, especially as they might give rise to linguistic markers of motor impairment.

### Language-Motor Rehabilitation

As mentioned earlier, experimental manipulations of language in healthy monolinguals (e.g., Aziz-Zadeh et al., 2006; Boulenger et al., 2009; Alemanno et al., 2012; Ghio et al., 2018) and bilinguals (see section Behavioral Studies and (Neuro-)physiological Studies) have been shown to impact the motor system. Conversely, experimental manipulations of the motor system in healthy monolinguals have been shown to impact lexico-semantic processing (e.g., Beilock et al., 2008; Glenberg et al., 2008; Willems et al., 2009; Locatelli et al., 2012; Tremblay et al., 2012; Bidet-Ildei et al., 2017; Vukovic et al., 2017;Gijssels et al., 2018). Moreover, experimental manipulations of the motor system in healthy bilinguals has been shown to impact visual perception of motor speech movements (e.g., Swaminathan et al., 2013). Importantly, no study has investigated the impact of experimental manipulations of the motor system on lexico-semantic processing in L2. Moving toward clinical studies, others examined the impact of experimental manipulations of the motor system in monolingual patients on lexico-semantic processing (e.g., dopaminergic treatment in Boulenger et al., 2008; motor training with dyslexic children in Trevisan et al., 2017).

With respect to neuromodulation interventions, transcranial direct current stimulation (tDCS) and TMS of the motor cortex of aphasic patients is of particular interest. While brain stimulation is increasingly being tested as promising auxiliary therapeutic tools in patients with aphasia, results have so far been inconsistent, the activation of different brain regions showing very different efficacy (Arévalo et al., 2007; Marangolo et al., 2016; see also Elsner et al., 2013; Lefaucheur et al., 2017 for reviews). The stimulation of the motor cortex is especially interesting considering that this region is easily located and it is often spared in aphasic patients (Branscheidt et al., 2017b; Dreyer and Pulvermüller, 2018). Recently, Branscheidt et al. (2017b) showed a specific role of the motor-cortex in accessing lexical-semantic content. Similarly, Meinzer et al. (2016) showed improved naming abilities after 2 weeks of concurrent speech and language therapy and left motor cortex stimulation. However, while these studies investigated effects of neuromodulation techniques on L1 processing, this question has not yet been addressed with bilingual patients. To the best of our knowledge, no clinical study has directly investigated the interaction between sensorimotor areas and L2.

With respect to behavioral interventions, we believe several methods to be relevant. Therapists can choose, for example, to reinforce the damaged language-specific neural network by training the specific language impairment or to work on a more general cognitive-control network reinforcing executive functions, or, in light of the studies on embodiment mentioned so far, strengthen the sensorimotor circuit. Several speech and language therapeutic approaches that are based on the interaction between the motor and the language systems, as in embodiment theories, have in fact shown promising results (e.g., Semantic Feature Analysis therapy, Boyle and Coelho, 1995; gestures production therapies, Krauss, 1998; Goldin-Meadow et al., 2001; Rose, 2006; Rose et al., 2013; Action Observation Therapy, Marangolo et al., 2010; language-action therapies, Difrancesco et al., 2012; Stahl et al., 2016). As an example, a motor recovery therapy based on the mirror neuron system, commonly called the Action Observation Therapy, has already been extended to the domain of aphasia. Marangolo et al. (2010) showed that after therapy, four non-fluent chronic lexico-phonological impaired aphasic patients improved in lexical retrieval as a result of both “action observation” therapy and “action observation and execution” therapy. Importantly, their improvement was still evident 2 months after the treatment. The authors suggested that the sensory-motor representations, activated by observing a performed action, served as input at the lexical level and facilitated word retrieval (Marangolo et al., 2010;Bonifazi et al., 2013). However, one other study showed no improvement in two aphasic patients with the same type of therapy, which was attributed to differences in the cognitive and linguistic profiles of the patients (Routhier et al., 2015). Nonetheless, Gili et al. (2017) – using fMRI – recently confirmed Marangolo et al.’s (2010) hypothesis by showing a sensorimotor recruitment following action observation therapy. They demonstrated a significant change in functional connectivity in the right sensorimotor networks when a significant linguistic improvement was present, suggesting that this therapy improves naming abilities in aphasic patients. Even more recently, Durand et al. (2018), explicitly attributed their rehabilitation approach (Personalized Observation, Execution, and Mental imagery therapy, POEM) to the recent evidence of the embodied framework and identified the neural substrate of their approach via neuroimaging before and after intervention. They combined the potential of action observation, gesture execution and mental imagery into the therapy of two aphasic patients (i.e., *proof of concept* study). Taking into account the preliminary nature of this study, the results showed a positive behavioral outcome for both trained and untrained items, and the neural changes were consistent with an account based on the interaction between the motor and the language systems. The potential of this kind of therapies is promising, yet requires further investigation including control interventions and relevant conditions to better identify the underlying mechanisms both in L1 and L2.

The Semantic Feature Analysis therapy (SFA, Boyle and Coelho, 1995), could also be considered as an experimental manipulation of the motor system, and may also be used in bilingual patients. Similarly to the Action Observation Therapy, the SFA therapy focuses on increasing the activation of semantic features (e.g., action, use, properties) associated with the target word to be retrieved. This intervention has shown a positive correlation between responsiveness to the therapy and the activation of the left precentral gyrus and the left inferior parietal lobule (Marcotte et al., 2012). The left inferior parietal lobule is a multimodal associative area, receiving auditory, visual and somatosensory input (Caspers et al., 2013), and connected to Wernicke’s and Broca’s areas via the arcuate fasciculus, a white matter tract passing through the precentral gyrus. Based on this, Durand and Ansaldo (2013) took the results from Marcotte et al. (2012) one step further and claimed this path to be recruited during Semantic Feature Analysis therapy, which can in turn lead to positive language production outcomes. For a recent review on the characteristics and effectiveness of SFA therapy results, see Efstratiadou et al. (2018). In terms of bilingualism, Knoph et al. (2015, 2017) were the only ones to measure the effect of SFA therapy in late acquired languages. The authors showed that an overall improvement in verb and narrative production in the treated language could be generalized to the untreated ones in multilingual speakers.

Finally, in regard to the issues mentioned so far, one does wonder whether experimental manipulations of the language system may also produce promising effects on the impaired motor system in monolingual and bilingual patients. Some studies do hint that this may be a promising line of research (e.g., Maitra et al., 2006). In Maitra et al. (2006), for example, patients that had suffered a stroke had their movements facilitated with self-speech (i.e., self-vocalization). As Anderlini et al. (2019) suggest, the choice of the type of therapeutic approach should consider both the language and motor systems and how they interact, especially when motor and language impairments coexist.

Of course, studies on L2 acquisition may be of special interest in future work on this topic too, as rehabilitation and learning may be grounded on similar mechanisms (e.g., motor areas response to learning the meaning of novel action words in Kiefer et al., 2007; Liuzzi et al., 2010; James and Swain, 2011; Fargier et al., 2012; Bechtold et al., 2018). Still, in sum, embodiment-based therapies offer interesting solutions in L1 and, given the data presented in this review, which assume language-motor association in both L1 and L2, potentially also in L2. In fact, bilingual rehabilitation, the cross language transfer (CLT) of treatment benefits from one language to the other(s) is a notable topic. It is not yet clear which factors influence the success of CLT in bilingual aphasics: premorbid language proficiency, degree and type of language impairments or various forms of therapy (Miertsch et al., 2009; Faroqi-Shah et al., 2010; Kiran and Iakupova, 2011; Kiran et al., 2013; Ansaldo and Saidi, 2014; Radman et al., 2016). Moreover, if the transfer does not take place, the selective recovery of one language could be seen as partial evidence of a different neural representation of the two languages. This issue though, has not yet been explored in the context of embodiment therapies. The engagement of (usually spared) motor areas and the knowledge about the degree of L1 and L2 embodiment could offer new hypotheses about CLT.

## The Future of L2 Embodiment Studies

### Theoretical Research

There are many challenging paths in this topic ahead of us, and for any rigorous attempt to better understand lexico-semantic embodiment in L2, we would suggest three critical issues to seriously consider. First, although all studies on the topic have concentrated on a *language-to-motor* directional effect, targeting a *motor-to-language* effect might improve our understanding of the language-motor interaction. This could be addressed by directly changing the excitability of the motor cortex with the application of non-invasive brain stimulation techniques and examining its impact on second language processing. The same goal can be addressed with lesion studies including bilingual patients with motor impairment or including elderly people. As sensory-motor and cognitive functions decline in aging (Baltes and Lindenberger, 1997), the reciprocal influence of these functions could be addressed in monolingual (Vallet, 2015) and bilingual elderly populations. Second, within-participant designs should be favored over between-participant ones. This is crucial in order to minimize the impact of inter-individual sociolinguistic differences, which have been shown to interact with language representations (e.g., De Groot, 1995). Third and finally – and closely related to the issue of processing stage discussed earlier – measurements and tasks enabling us to specify both space and time characteristics of the mechanisms under investigation should be carefully chosen. For example, functional neuroimaging tools, may provide us with both strength and timing (i.e., onset and duration) of any sensorimotor activation, given they are used in conjunction with the appropriate tasks. More specifically, these tasks should enable us to appropriately access both shallow and deep processing (e.g., lexical and semantic access).

### Clinical Research

We believe that this shift in treatment approaches – merging traditional speech and language therapies with a motor integrated perspective – opens new directions in bilingual aphasia rehabilitation. We argue, though, that three necessary issues need to be further addressed and clarified in future studies. First, due to the scarce literature on the subject, additional pre-registered and randomized controlled studies need to be conducted to confirm that therapies based on sensorimotor activation do indeed improve L1 language processing, specifically for sensorimotor-related stimuli in aphasics. Second, clear evidence needs to be provided to show that the same therapy can improve L2 language processing, again, specifically for sensorimotor-related stimuli in aphasics. To our knowledge, only Knoph et al. (2015, 2017) have provided SFA therapy in L2, providing some evidence of improvement in L2. Third and finally, given additional evidence corroborating Knoph and colleagues’ findings, therapy outcomes in L2 and L1 would need to be compared and contrasted. Typically, a crossover randomized control trial study could be conducted to address this, provided that the factors influencing therapy outcomes in L1 and L2 (e.g., language competence) are taken into account. Theoretically, it will also bring further enlightenment on differences of the degree of L2 embodiment compared to L1. Clinically, it will bring evidence-based driven awareness in the choice of the therapeutic approach and the language of the therapy. Given that these three issues are rigorously addressed, it should enable us to directly focus on the CLT of therapy outcomes. More specifically, the direction (i.e., L1 to L2, L2 to L1, or both) and magnitude of the transfer could provide us with new insights into the mechanisms underlying embodiment effects. Importantly, we argue that *embodied* therapies could well complement conventional ones – not supplant them –, both still needing more data for clinicians to choose and apply evidence-based interventions.

## Conclusion

In light of the exponential increase in multilingual populations worldwide, a better understanding of the mechanisms underlying the interplay between neural structures involved in the processing of more than one language is central. The sensorimotor embodiment account offers an opportunity to further our knowledge in several areas of research, including semantic processing in mono- and bilinguals, language learning, neural mechanisms of language processing and rehabilitation in L2. Overall, all the reviewed studies investigating sensorimotor involvement in semantic processing showed that L2 is – at least to some extent – embodied. Further investigating the factors influencing the degree of L2 embodiment is relevant from a theoretical point of view, of course, but also to confirm or dismiss the value of language therapeutic approaches based on embodiment theories as a complement of speech and language therapies in bilinguals. We have outlined several important issues to tackle in the future, and hope that these will be taken as a sign to encourage rigorous and innovative research in this topic, both in a theoretical and applied perspective.

## Author Contributions

All authors listed have made a substantial, direct and intellectual contribution to the work, and approved it for publication.

## Conflict of Interest Statement

The authors declare that the research was conducted in the absence of any commercial or financial relationships that could be construed as a potential conflict of interest.

## Footnotes

1 Taylor et al. (2017) argued that in those studies, action and motion were not considered separately, leading to erroneous interpretations.

2 Other examples of studies on L2 acquisition and embodiment are provided in the Section “Critical Synthesis.”

3 Here we do not discuss the case of bicultural bilinguals, but reader**s** can refer to Jared et al. (2013) and Adams (2016) for a discussion on this subject.

4 Cognates are words that share orthographic and/or phonologic features between languages (e.g., *nemen* in Dutch with *nehmen* in German [to take]).
